# SPAID: a comprehensive database for disease-specific autoantigens in autoimmune disorders

**DOI:** 10.1007/s44307-026-00117-8

**Published:** 2026-06-08

**Authors:** Shunhui Deng, Fangfang Wei, Ya Pang, Luowanyue Zhang, Shengyao Zhi, Tianjian Chen, Zhixiang Zuo, Jian Ren, Yubin Xie, Xiaotong Luo

**Affiliations:** 1 Innovation Center of the Sixth Affiliated Hospital, School of Life Sciences, Sun Yat-Sen University, Guangzhou, 510060 China; 2 Department of Infectious Disease, The Sixth Affiliated Hospital, Sun Yat-Sen University, Guangzhou, 510655 Guangdong China; 3 Guangdong Provincial Key Laboratory of Pharmaceutical Bioactive Substances, School of Biosciences and Biopharmaceutics, Guangdong Pharmaceutical University, Guangzhou, 510006 China; 4 State Key Laboratory of Oncology in South China, Cancer Center, Collaborative Innovation Center for Cancer Medicine, Sun Yat-Sen University, Guangzhou, 510060 China; 5 Institute of Precision Medicine, The First Affiliated Hospital, Sun Yat-Sen University, Guangzhou, 510060 China; 6 Biomedical Innovation Center, Guangdong Institute of Gastroenterology, The Sixth Affiliated Hospital, Sun Yat-Sen University, Guangzhou, 510060 China

**Keywords:** Autoimmune diseases, Proteomics, Non-canonical proteins, Small peptides, Candidate autoantigens

## Abstract

Autoimmune diseases (ADs) are chronic inflammatory disorders characterized by complex etiologies and significant diagnostic challenges. Although autoantigens are critical for precision diagnosis and therapy, much of the immunogenic landscape remains unexplored due to the historical focus on canonical proteins. Here, we developed SPAID (https://spaid.renlab.cn), a comprehensive resource for candidate autoantigen discovery across 14 ADs that integrates canonical and non-canonical proteins within a two-level evidence framework. The validated level contains proteins associated with experimentally confirmed epitopes from T-cell assays and major histocompatibility complex (MHC) ligand assays. The proteomics-based level contains proteins identified by mass spectrometry (MS) from human samples, further annotated with differential expression patterns, immunogenicity scores, and functional features to support candidate autoantigen discovery and further validation. By combining validated evidence with proteomics-based evidence, SPAID enables the comprehensive characterization of candidate autoantigen repertoires and facilitates mechanistic investigation into antigen origins and pathogenic recognition. Overall, SPAID provides a foundational resource for advancing antigen-centered research and developing novel diagnostic and therapeutic strategies in autoimmunity.

**Supplementary Information:**

The online version contains supplementary material available at 10.1007/s44307-026-00117-8.

## Introduction

Autoimmune diseases (ADs) arise from a breakdown of immune tolerance to self-proteins, leading to sustained immune responses against endogenous antigens (Singh et al. 2016). Protein autoantigens are central to this process because they directly initiate and maintain autoreactive T-cell and B-cell activation. Understanding their origins, biochemical properties, and immunogenic features is essential for clarifying disease mechanisms and for developing precise diagnostic and therapeutic strategies. However, despite extensive studies on genetic susceptibility (Gutierrez-Roelens et al. 2008), environmental triggers (Gulati et al. 2018), immune dysregulation (Yurasov et al. 2005), and autophagy-related pathways (Lin et al. 2026; Yuan et al. 2024), the mechanisms that generate pathogenic autoantigen epitopes remain insufficiently defined, and the landscape of disease-associated autoantigens is still incomplete.

Recent evidence indicates that autoantigens can originate not only from conventional protein-coding genes but also from proteins translated from non-coding regions, including non-coding RNAs (ncRNAs) and introns (Nguyen et al. 2021; Starck et al. 2016). These non-canonical proteins may be aberrantly expressed under inflammatory or immune-stressed conditions and presented as novel immunogenic peptides (Lodha et al. 2022). Their restricted expression patterns and potential disease specificity suggest that they may serve as sensitive indicators of early autoimmune activation and offer mechanistic insights beyond those provided by classical autoantigens. Systematic characterization of both canonical and non-canonical proteins is therefore critical for identifying pathogenic epitopes and improving disease stratification.

Existing databases catalog AD-related genes or established autoantigens, but they focus primarily on classical protein-coding genes and rarely incorporate proteome-wide MS evidence. The Autoimmune Disease Database lists over 1,200 disease names and extracts associated genes and proteins (Karopka et al. 2006). AAgAtlas provides browsing, search, and download functions for human autoantigens (Wang et al. 2017). Autoimmune Disease Explorer integrates gene expression and methylation data from five ADs (Martorell-Marugán et al. 2021). PGG.MHC aggregates human leukocyte antigen (HLA) gene information linked to phenotypes of autoimmune, infectious, oncologic, and psychiatric disorders (Zhao et al. 2023). Current databases primarily focus on gene-centric annotations, while proteome-level characterization of ADs and proteome-informed autoantigen discovery remain underrepresented. This limitation underscores the need for resources that systematically integrate proteomic evidence across ADs.

**Fig. 1:**
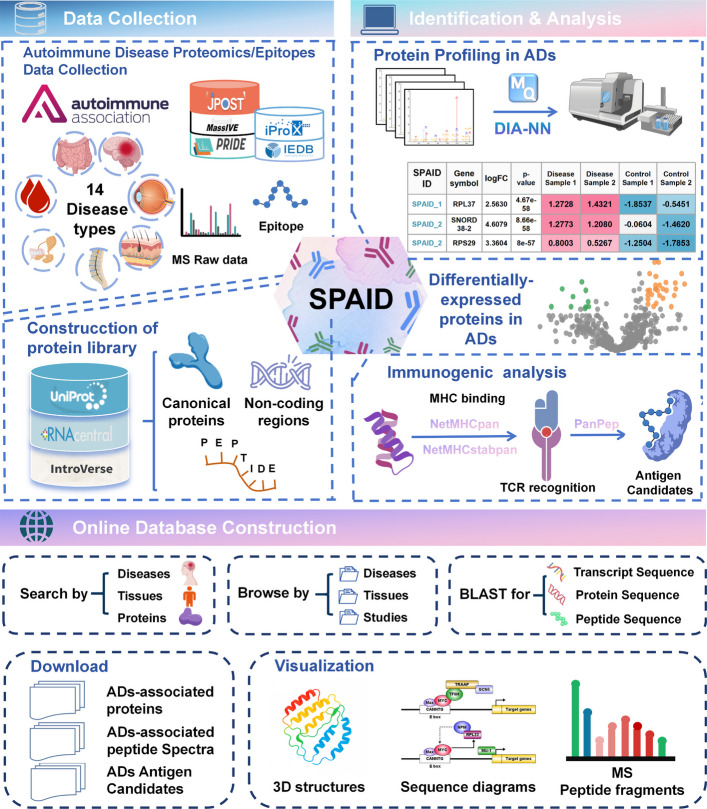
Overall design and construction of SPAID. From published literature and available databases, we collected ~ 176,363 peptide sequences from ~ 51 proteomic cohorts across 14 autoimmune diseases, corresponding to ~ 26,085 proteins of various types in SPAID. In addition, the workflow incorporated 21,565 experimentally validated epitopes associated with 21,349 proteins (upper left). MaxQuant and DIA-NN were applied to search for peptides in the collected proteomic datasets. Protein expression was quantified by aggregating the normalized intensities of corresponding peptides. Differential expression analysis was then performed at the protein level between disease and control samples. To identify potential autoantigens, SPAID used a robust pipeline to assess the immunogenicity of AD-associated proteins (upper right). Besides, SPAID provides a comprehensive online platform for exploring AD-associated proteins. Users can search or browse by various criteria such as disease or tissue, and perform BLAST with transcript, protein, or peptide sequences. Data can also be downloaded, including AD-associated proteins, peptide spectra, and antigen candidates. Additional visualization tools are provided for 3D structures, sequence diagrams, and MS-derived peptides (lower). The diagram was created with BioGDP.com (Jiang et al. 2025)

To address these gaps, we developed SPAID (https://spaid.renlab.cn), a platform for systematic candidate autoantigen discovery in ADs that captures canonical and non-canonical proteins (Fig. 1). SPAID organizes evidence into two distinct levels. The validated level includes proteins with literature-curated epitopes supported by experimental immunological evidence from T-cell and MHC ligand assays. The proteomics-based level contains disease-associated peptides and proteins identified by MS from human samples across 14 ADs. To facilitate candidate autoantigen discovery, the entries from the proteomics-based level are systematically annotated with differential expression patterns, immunogenicity scores, and functional features. As a demonstration of practical utility, we leveraged SPAID to analyze an independent rheumatoid arthritis (RA) cohort. SPAID effectively verified known biomarkers, discovered novel marker candidates, and uncovered potential autoantigens to guide downstream functional validation.

By integrating validated and proteomics-based evidence, SPAID provides a comprehensive landscape of canonical and non-canonical candidate autoantigens. This platform serves as a critical resource to accelerate mechanistic research and target discovery in ADs.

## Materials and methods

### Overall design and framework of SPAID

SPAID provides a systematic, disease-centric platform for organizing protein-level candidate autoantigen evidence across various ADs. We initially compiled a list of ADs from the Autoimmune Association (https://autoimmune.org). Based on a manual literature review of pathogenetically implicated autoantigens, we curated 14 distinct ADs into SPAID.

Based on data origin, SPAID organizes candidate autoantigen evidence into two distinct levels. The validated level comprises proteins with literature-curated epitopes supported by experimental evidence from T-cell and MHC ligand assays. On the other hand, the proteomics-based level encompasses disease-associated peptides and proteins identified via MS from human samples. To standardize and integrate these diverse data types, SPAID maps all entries onto a unified protein sequence space serving as a common reference framework.

### *Co*nstruction of the unified protein sequence space

To build the unified protein sequence space, we curated candidate sequences from human ncRNA (RNAcentral) (The RNAcentral Consortium 2019) and intronic regions (IntroVerse) (García-Ruiz et al. 2023). Coding potential was evaluated using CPAT (Wang et al. 2013) and CNCI (Sun et al. 2013), and only sequences meeting both thresholds were retained (CPAT score > 0.364; CNCI score > 0). Open reading frames (ORFs) were then predicted via NCBI’s ORFfinder (Sayers et al. 2023) and translated into amino acid sequences. To remove redundancy, predicted non-canonical proteins identical to reviewed human proteins in UniProt (UniProt Consortium 2023) were excluded. Finally, the remaining unique candidates were merged with the UniProt reference set to construct a unified protein sequence space for validated epitope mapping and MS-based protein identification.

### Construction of the two-level evidence framework

The two-level evidence framework was constructed by curating immunological and proteomic evidence from divergent streams.

For the validated level, experimentally validated epitopes associated with the 14 ADs were curated from the Immune Epitope Database (IEDB) (Vita et al. 2025) using disease-specific query strategies. To ensure maximum specificity, each disease was queried individually using exact search terms rather than broad hierarchical categories. The dataset was stringently filtered to include only linear peptides with positive outcomes in T-cell or MHC ligand assays. Regarding taxonomic constraints, source species were restricted to *Homo sapiens*, whereas host organisms included both *Homo sapiens* and *Mus musculus*, with no restrictions applied to MHC alleles. For each epitope, we extracted its assay types and source protein along with the supporting literature. All epitopes were then mapped to the unified protein sequence space, with matching proteins defining the validated level of SPAID.

The proteomics-based level was constructed by analyzing public proteomic datasets (Sects. 2.4 to 2.8). Following an optimized workflow adapted from Othoum et al. (Othoum et al. 2023), to minimize false positives, public datasets were curated and searched against the reference library with stringent false discovery rate (FDR) control. Protein abundance was estimated from peptide intensities for differential expression analysis, while immunogenicity was evaluated at both the peptide and protein levels. In addition, these data were comprehensively annotated by incorporating diverse functional, structural, and translational dimensions.

### Collection of autoimmune disease-related proteomic datasets

Proteomic datasets were systematically retrieved from public repositories, including PRIDE (Perez-Riverol et al. 2025), MassIVE.quant (Choi et al. 2020), jPOST (Moriya et al. 2019), PeptideAtlas (Deutsch et al. 2008), and iProX (Chen et al. 2022), using disease-specific search terms. Human datasets published before December 2024 and containing both disease and matched control samples were retained.

### MS-based peptide identification and protein quantification

Peptides were identified by searching MS/MS spectra against the reference library. To mitigate the high false-positive risk associated with non-canonical peptides, stringent FDR control and conservative filtering were applied at the peptide-spectrum match (PSM), peptide, and protein-group levels.

For data-independent acquisition (DIA) datasets, raw files were processed via DIA-NN 2.0 (Demichev et al. 2020), generating dataset-specific spectral libraries in library-free mode (– gen-spec-lib) against the unified protein sequence space. Digestion enzymes and study-specific parameters matched the original experimental designs. Search constraints included a peptide length of 7–30 amino acids, precursor m/z range 300–1800, fragment m/z range 200–1800, precursor charges 1–4, and enabled N-terminal methionine excision. Precursor and protein-group FDRs were strictly controlled at 1% (– qvalue 0.01). Robustness was enhanced using re-analysis (– reanalyse), retention-time profiling (– rt-profiling), and parsimonious protein-group inference (– pg-level 1).

For data-dependent acquisition (DDA) datasets, raw files were searched using MaxQuant 2.6.5 (Tyanova et al. 2016). Parameters were aligned with study-specific designs, setting carbamidomethyl (C) as a fixed modification, alongside variable oxidation (M) and N-terminal acetylation. Identification required a minimum peptide length of 7 amino acids and a 1% FDR for both PSMs and protein groups. Modified peptides further required an Andromeda score ≥ 40 and a delta score ≥ 6. Second-peptide search was enabled, with all other parameters left at defaults.

Extracted peptide intensities were normalized using platform-specific protocols, including precursor-level normalization for DIA, MaxQuant’s delayed normalization for label-free DDA, and reference-channel scaling for labeled data (TMT, iTRAQ, SILAC, dimethyl).

For protein quantification, peptides were allocated based on a canonical-priority strategy. Peptides with canonical matches were assigned exclusively to those canonical proteins, whereas those without canonical matches were distributed to all matching non-canonical proteins without intensity splitting. Protein abundance was defined as the mean normalized intensity of all assigned peptides.

### Differential expression analysis

For each collected dataset, samples were stratified by disease subtype and tissue type to construct distinct comparison cohorts for differential expression analysis. Each cohort paired a specific disease subtype group with a matched control group from the same tissue type. Protein abundances were ${\mathrm{l}\mathrm{o}\mathrm{g}}_{2}$ transformed within each cohort to stabilize variance and improve normality. Differential expression between disease and control groups was subsequently assessed by a two-sample t-test. ${\mathrm{l}\mathrm{o}\mathrm{g}}_{2}$ fold change (${\mathrm{l}\mathrm{o}\mathrm{g}}_{2}$ FC) was calculated as the difference in mean ${\mathrm{l}\mathrm{o}\mathrm{g}}_{2}$-transformed abundance between groups. Proteins with p < 0.01 and |${\mathrm{l}\mathrm{o}\mathrm{g}}_{2}$ FC|> 0.58 were considered significantly differentially expressed, with positive and negative ${\mathrm{l}\mathrm{o}\mathrm{g}}_{2}$ FC values indicating upregulation and downregulation, respectively. Proteins detected only in disease samples were classified as ‘disease-only detected’ for that cohort.

For ADs with multiple available cohorts, protein-level results were integrated across studies. Within each cohort, proteins were ranked by ${\mathrm{l}\mathrm{o}\mathrm{g}}_{2}$ FC, and Robust Rank Aggregation (RRA) (Kolde et al. 2012) was applied to evaluate the congruence of these rankings. RRA scores quantified cross-cohort stability, where lower scores indicated greater consistency. By synthesizing cohort-level differential expression and disease-level RRA results, we assigned proteins to four categories: (1) Disease-only detected: Proteins uniquely identified in patient samples relative to controls in at least one cohort and upregulated in other cohorts, with RRA score < 0.5. (2) Upregulated and (3) Downregulated: proteins consistently showing positive or negative trends across studies, with RRA score < 0.5; and (4) Other: all remaining proteins.

### Immunogenicity prediction for mass spectrometry-derived proteins

To identify candidate autoantigens, we performed immunogenicity predictions on peptides from our AD-related proteomic datasets using an established pipeline (Luo et al. 2022). This framework evaluates antigen presentation by integrating three core metrics: MHC binding affinity, peptide-MHC stability, and T-cell recognition probability (Wells et al. 2020).

To identify potential epitopes, MS-identified peptides were segmented into overlapping 8- to 14-mer fragments, with a focus on HLA class I (HLA-I) molecules. HLA-I alleles were classified into 12 distinct functional supertypes based on the physicochemical properties of their binding grooves (Wang et al. 2014). Since patient-specific HLA-I genotypes could not be directly inferred due to the lack of genomic or transcriptomic sequencing data, we employed functional conservation within HLA supertypes, in which alleles share overlapping peptide-binding motifs. Immunogenic potential was consequently evaluated using a representative prototype allele from each of the 12 major HLA-I supertypes: HLA-A*01:01, A*02:01, A*03:01, A*24:02, A*26:01, B*07:02, B*08:01, B*15:01, B*27:05, B*39:01, B*40:01, and B*58:01. For ADs with documented genetic associations, such as ankylosing spondylitis and psoriasis, we supplemented these analyses with known risk alleles, including HLA-B*27 subtypes (:05,:04, and:02) (Khan 2013) and HLA-C*06:02 (Prinz 2018). A peptide was deemed an 'immunogenic candidate' if any fragment satisfied all three criteria under at least one HLA-I subtype.

These candidates underwent further refinement via PanPep (Gao et al. 2023) to assess T-cell receptor (TCR) interactions. By calculating interaction probabilities against 419 complementarity-determining region 3 (CDR3) sequences, we designated peptides with a PanPep score > 0.7051 (FDR < 0.05) as 'immunogenic-positive.' Finally, to construct a disease-level antigenic landscape, we aggregated these peptide-level findings at the protein level. We classified a protein as a candidate autoantigen if it harbored at least one 'immunogenic-positive' MS-detected peptide. This information was subsequently incorporated into SPAID at the proteomics-based level.

### Functional, structural, and translational annotation of proteins in SPAID

Functional, genomic, and transcript-level annotations for the identified proteins were compiled from multiple repositories, including UniProt, RNAcentral, IntroVerse, CCDS (Pujar et al. 2018), Ensembl (Dyer et al. 2025) and RefSeq (O'Leary et al. 2016). Three-dimensional (3D) protein structures were obtained from experimentally determined PDB files retrieved via UniProt. For proteins lacking experimental structures, including both canonical and non-canonical isoforms, structural predictions were generated using ColabFold (v1.5.5) (Mirdita et al. 2022). Additionally, potential post-translational modification (PTM) sites were predicted across the proteome using PTM-Mamba (Peng et al. 2025).

In addition, to assess translational evidence supporting the translation of non-canonical proteins identified in SPAID, we systematically reviewed published literature for experimental evidence. A targeted PubMed search was conducted using keyword combinations such as “(ncRNA) AND (translation)” and “(intron) AND (translation),” yielding 23,156 candidate publications. Following a manual full-text review, only proteins with direct experimental evidence of peptide-level translation were curated and annotated as validated non-canonical translation products.

### Integration and database construction

All metadata within SPAID was integrated and managed in a MySQL relational database. The system architecture comprises a Java-based backend and a responsive multi-page frontend built with HTML, CSS, and JavaScript, featuring interactive data visualizations powered by ECharts. The entire platform is deployed on the Amazon Web Services (AWS) EC2 environment. SPAID provides a user-friendly web interface that facilitates the searching, browsing, and downloading of proteins, candidate autoantigens, and epitopes.

### Proteomic profiling of Rheumatoid Arthritis

This study was approved by the Ethics Committee of the Sixth Affiliated Hospital of Sun Yat-sen University, with written informed consent obtained from all participants. Residual serum samples from 5 RA patients and 5 healthy controls (HCs) were stored at − 80 ℃, cleared (12,000 × g, 10 min, 4 ℃), and depleted of the top 14 abundant proteins using the Pierce™ Top 14 Kit (Thermo Fisher Scientific). Following BCA quantification, proteins were reduced (5 mM dithiothreitol, 56 ℃, 30 min), alkylated (11 mM iodoacetamide, room temperature, dark, 15 min), diluted to < 2 M urea with 100 mM TEAB, and sequentially digested with trypsin at 1:50 overnight and 1:100 for 4 h. Peptides in solvent A (0.1% FA, 2% ACN) were separated at 500 nL/min on an in-house packed reversed-phase column (25 cm × 100 μm i.d.) via an EASY-nLC 1200 system using a multi-step solvent B (0.1% FA in 90% ACN) gradient: 7–20% (0–16 min), 20–32% (16–24 min), 32–80% (24–27 min), and 80% (27–30 min). An Orbitrap Exploris 480 MS with a nano-ESI source (2.3 kV) and FAIMS (− 45/− 70 V) was used for analysis. Full MS (m/z 390–810) and HCD MS/MS (fixed first mass m/z 200) scans were acquired at 30,000 resolution, utilizing NCEs of 25%/30%/35%, an AGC target of 3 × 106, and automated injection time. Raw DIA data were processed per Sects.  2.5 and 2.6.

## Results

### Overview and content of SPAID

Across 14 ADs, SPAID systematically integrates candidate autoantigen evidence by classifying data into two distinct tiers, designated as the validated level and the proteomics-based level. To standardize and unify the diverse data types underlying these two levels, the platform establishes a non-redundant, unified protein space of 576,516 sequences as its reference framework. This shared library integrates 42,444 canonical UniProt proteins with 534,072 non-canonical proteins. To construct this non-canonical repertoire, we collected 660,264 ncRNA sequences from RNAcentral and 332,571 intronic sequences from IntroVerse, evaluated their coding potential, and predicted open reading frames. Following translation and redundancy removal, we ultimately retained 447,445 intron-derived and 86,627 ncRNA-derived proteins.

**Fig. 2:**
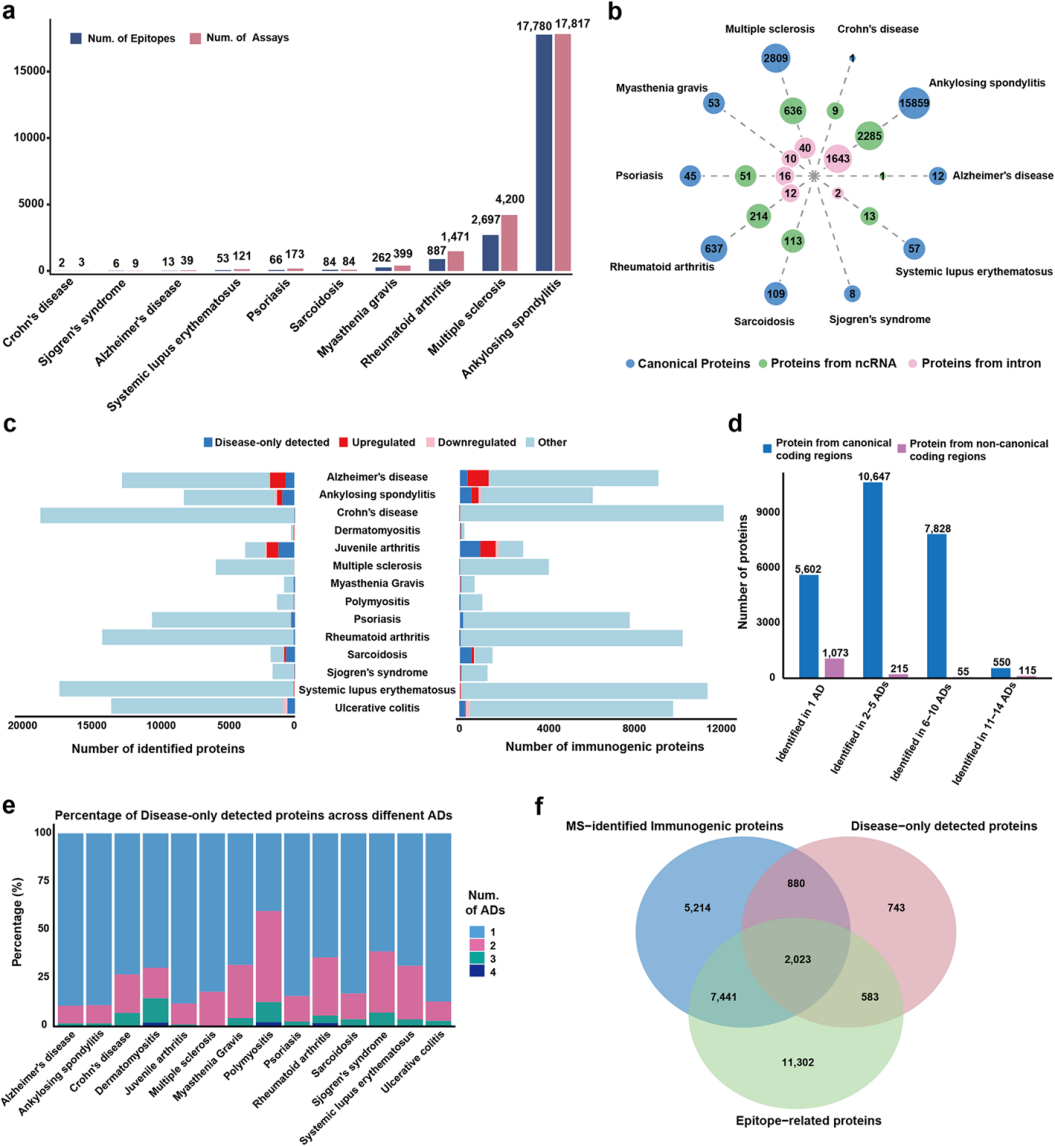
Overview of the data in SPAID. **a**: Number of experimentally validated epitopes and corresponding assays. Blue bars represent the number of epitopes, and pink bars represent the number of assays. **b** Bubble plot illustrating the number of epitope-related proteins associated with various ADs, with different colors representing protein types: canonical proteins (blue), proteins from ncRNA (green), and proteins from introns (pink). The size of each bubble indicates the number of proteins of that type associated with each autoimmune disease. **c** The number of all MS-identified proteins (left) and predicted immunogenic proteins (right) identified by SPAID in different ADs is shown in bar plots, with four expression groups: disease-only detected (deep blue), up-regulated in diseases (red), down-regulated in diseases (pink), and others (light blue). **d**: The bar plot shows the number of the two major protein types identified across multiple ADs. **e**: The bar plot illustrates the proportion of disease-only detected proteins that are additionally detected as specific in multiple other diseases. **f**: Venn diagram illustrates the overlap among MS-identified immunogenic, disease-only, and epitope-related proteins

For the validated level, we integrated T-cell and MHC ligand assay data from 292 publications, yielding over 20,000 validated epitopes. Exact sequence matching against the reference library identified 1,141 unique epitopes from 10 ADs, supported by 2,750 positive T-cell assay records. Similarly, we identified 20,424 distinct epitopes from 21,566 positive MHC ligand assays across 5 ADs (Fig. 2a). In total, these epitopes mapped to 21,349 unique proteins in the reference library, including 16,966 canonical proteins, 1,681 intron-derived proteins, and 2,702 ncRNA-derived proteins (Fig. 2b).

The proteomics-based level included 675 human proteomic samples from 14 ADs, which were manually stratified into 51 cohorts (Table S1). After database searching and protein quantification, 176,363 MS-identified peptides were assigned to 26,085 disease-associated proteins, including 927 ncRNA-derived proteins supported by 541 unique peptides and 531 intron-derived proteins supported by 457 peptides. These MS-identified proteins were further annotated using differential expression analysis, immunogenicity prediction, and functional features to facilitate systematic characterization and discovery of candidate autoantigens.

### Protein expression patterns across autoimmune diseases

Based on the differential expression analysis within each study, proteins were classified into four distinct expression groups according to the criteria detailed in the Methods Sect. 2.6. Across the 14 ADs, we identified 4,577 ‘disease-only detected’ proteins, 2,571 significantly upregulated proteins, and 757 significantly downregulated proteins (Fig. 2c, left panel). Non-canonical proteins were also represented, including 193 ncRNA-derived and 28 intron-derived proteins in the ‘disease-only detected’ group, as well as 9 showing significant upregulation. We next examined the extent to which ‘disease-only detected’ proteins were shared across ADs (Fig. 2e). Although most proteins were disease-specific, a subset was still shared across conditions. For example, 47.37% (27/57) of ‘disease-only detected’ proteins in polymyositis were also identified as disease-specific in at least one other AD. Similar patterns, with overlap rates of approximately 30%, were observed in myasthenia gravis (MG), RA, Sjögren’s syndrome (SS), and systemic lupus erythematosus (SLE). This widespread sharing firmly links these proteins to generalized immune dysregulation, pointing to their roles as potential drivers of pathogenesis.

Among the 1,458 non-canonical proteins identified across 14 ADs, 26.41% (385/1,458) were shared across multiple diseases (Fig. 2d). To evaluate their novelty and reliability, we cross-referenced these sequences with existing translational evidence from published literature. Only 1.10% (16/1,458) had been previously supported by MS, peptide-tagging, or antibody-based studies and were therefore labeled as “validated” in SPAID, indicating that the majority represent previously unrecognized translation products. Given this limited literature support, we evaluated the robustness of these proteins by assessing their reproducibility within diseases supported by at least three independent proteomic cohorts (Table 1). In four of the five included ADs, over 40% of the non-canonical proteins were repeatedly detected across independent cohorts: psoriasis (61.51%, 171/278), SLE (54.58%, 280/513), Crohn's disease (51.41%, 384/747), and RA (40.32%, 150/372). Although SS exhibited minimal reproducibility (0.79%, 1/127) due to confounding biofluids and low detection depth, the remaining diseases showed remarkable consistency in the expression of reproducibly identified proteins. This trend was particularly prominent in RA (99.33%, 149/150), SLE (93.93%, 263/280), and psoriasis (87.72%, 150/171). This high concordance in both identification frequency and expression patterns strongly demonstrates that these signals represent robust, disease-specific biological entities rather than random MS noise.

**Table 1:** Reproducibility of non-canonical protein identification and expression patterns

Disease	Number of cohorts^a^	Number of non-canonical proteins^b^	Reproducibly identified proteins^c^	Reproducibility ratio (%)^d^	Expression-consistent proteins^e^	Expression consistency ratio (%)^f^
Psoriasis	3	278	171	61.51	150	87.72
Systemic lupus erythematosus	12	513	280	54.58	263	93.93
Crohn's disease	11	747	384	51.41	193	50.26
Rheumatoid arthritis	9	372	150	40.32	149	99.33
Sjögren's syndrome	5	127	1	0.79	1	100

^a^Number of independent proteomics cohorts available for each disease.

^b^Total number of unique non-canonical proteins identified across all cohorts for each disease.

^c^Number of non-canonical proteins identified in more than one independent cohort for the same disease.

^d^Percentage of reproducibly identified proteins among all identified non-canonical proteins for a given disease. Calculated as: (Reproducibly identified proteins/Number of non-canonical proteins) × 100.

^e^Number of reproducibly identified proteins whose expression group was identical across all cohorts within the same disease.

^f^Percentage of expression-consistent proteins among all reproducibly identified proteins for a given disease. Calculated as: (Expression-consistent proteins/Reproducibly identified proteins) × 100.

### Identification of candidate autoantigens

To screen the extensive proteomic data for candidates capable of triggering immune responses, we evaluated the immunogenicity of 176,363 detected peptides (Methods 2.7). Overall, 14.70% (25,918) of these peptides were classified as putatively immunogenic, with the highest proportions observed in MG (21.93%) and SS (20.83%). Validating this strategy, 37 predicted immunogenic peptides were independently supported by MHC ligand assay data (Table S2).

Mapping these peptide-level predictions back to their source proteins identified 15,558 immunogenic proteins, accounting for 59.64% of all disease-associated proteins in SPAID (Fig. 2c, right panel). We next examined whether this predicted immunogenicity was preferentially associated with proteins showing disease-related expression changes. Notably, immunogenic candidates were highly enriched among 'disease-only detected' proteins, representing 69.24% (3,196) of this subset. This group comprised 3,053 canonical and 118 non-canonical proteins, the latter consisting of 109 ncRNA-derived and 7 intron-derived sequences. Furthermore, predicted immunogenic proteins were more frequent in the upregulated group than in the downregulated group (86.43% vs. 78.01%). This enrichment likely reflects that proteins elevated under inflammatory conditions contribute more robustly to the substrate pool for antigen processing and presentation. In this context, tissue damage and inflammatory environments may facilitate the presentation of otherwise sequestered self-determinants, including cryptic epitopes (Vanderlugt et al. 2002).

To define a set of high-confidence candidate autoantigens, we integrated three features: disease-specific proteomic detection, predicted immunogenicity, and experimental epitope support. The intersection of these three features identified a core set of 2,023 proteins (Fig. 2f). These proteins represent the most promising candidates within the SPAID database, as they are simultaneously associated with disease states, supported by known epitopes, and characterized by high immunogenic potential. Collectively, these findings highlight the value of integrating proteomic discovery with immunogenicity filtering to better characterize both canonical and non-canonical drivers of autoimmune responses.

### Web interface and usage

**Fig. 3:**
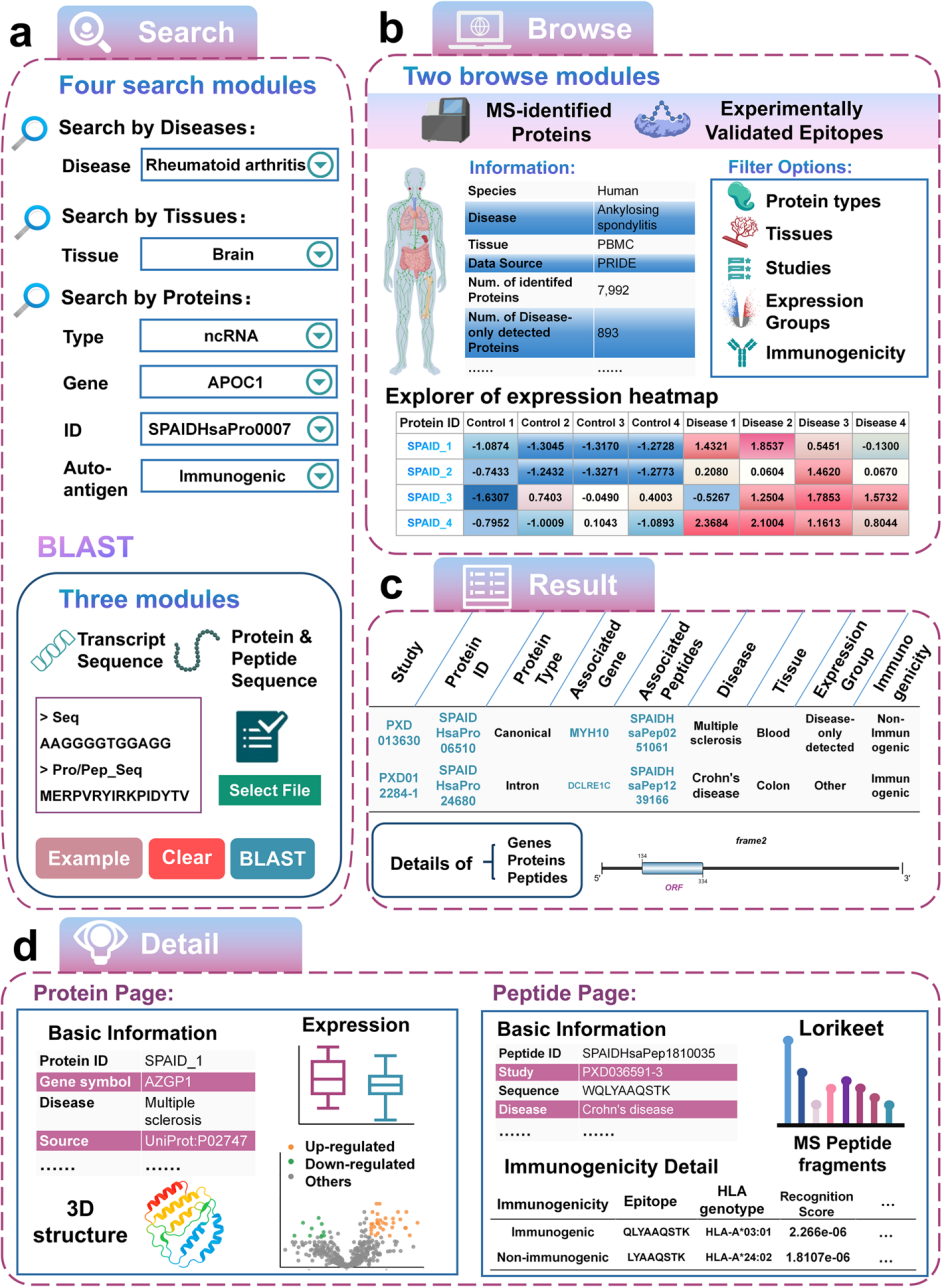
Basic functions of SPAID web interface. **a**: The main modules of the search interface of SPAID and the BLAST interface for sequence search. **b**: The browsing interface in SPAID, which is divided into two modules: MS-identified proteins and experimentally validated epitopes. **c**: The result module in SPAID is divided into three levels, including gene level, protein level, and peptide level. **d**: Detailed views of the protein and peptide page interfaces in SPAID

To ensure the utility of SPAID for the research community, we developed a user-friendly web interface that integrates all curated genomic, proteomic, and epitope data. The platform’s functionalities begin with a multimodal search engine and a customized BLAST suite (Fig. 3a). The search interface is categorized into four primary modules, allowing users to query by disease type, tissue origin, or specific protein attributes such as gene symbols, identifiers, and immunogenicity status. Complementing this, the integrated BLAST tool allows users to perform similarity searches by submitting query sequences against three comprehensive internal datasets: transcripts, proteins, and peptides, with full parameter customization to support tailored comparative analysis.

Following the initial query, the Browse page serves as a dual-level entry point for exploring the autoimmune landscape at both the protein and epitope levels (Fig. 3b). In the MS-identified Proteins view, users can navigate specific disease and tissue categories to access a comprehensive statistical overview, including the total number of identified proteins and those detected exclusively in disease states. This view features an expression heatmap explorer that visualizes protein abundance across multiple disease and control samples, facilitating the rapid identification of differential expression patterns. Alternatively, the Experimentally Validated Epitopes view enables the exploration of 24,316 experimental records, allowing users to toggle between T-cell and MHC ligand assay categories to examine evidence for epitope-disease associations. All resulting data are then presented in a structured table that integrates multi-omics layers, displaying the expression group and predicted immunogenicity for each entry. An interactive filter panel at the bottom of the table enables users to refine large datasets (Fig. 3c).

To provide a granular view of the identified candidates, SPAID organizes detailed annotations into three hierarchical levels across dedicated Protein, Peptide, and Gene pages (Fig. 3d). The Protein page integrates basic identifiers with expression boxplots, volcano plots, and 3D structural visualizations derived from either PDB or ColabFold, alongside mapped post-translational modification sites. Also, to intuitively illustrate the locations of ORFs within non-canonical transcripts, SPAID employs IBS 2.0 (Xie et al. 2022) to generate schematic diagrams. For deeper evidence validation, the Peptide page provides sequence-specific details, HLA-specific immunogenicity scores, and a Lorikeet-powered spectrum annotator for MS/MS fragment visualization. Each record is cross-referenced with external identifiers such as IEDB and PubMed IDs, while bidirectional mapping ensures that users can easily transition between experimental epitope evidence and proteomic validation. Finally, all structured datasets, the unified protein sequence space and experimental epitope records, are made available via a dedicated Download page for academic use, supplemented by a comprehensive Help section for step-by-step guidance.

### SPAID enables the discovery of disease-specific biomarkers in rheumatoid arthritis

To validate SPAID for biomarker discovery, we analyzed an independent serum proteomics dataset comparing five RA patients with five HCs (Fig. S1a). Of the 1,559 identified proteins (Table S3), 132 formed our RA-associated set, including 122 (7.8%) RA-specific, 4 (0.26%) significantly upregulated, and 6 (0.38%) significantly downregulated proteins (Fig. S1b).

We applied a two-step validation pipeline via SPAID to filter clinical biomarkers from this candidate set. First, an overlap analysis with external RA proteomics datasets curated in SPAID revealed 23 proteins. This subset highlighted immune-related factors such as gamma-interferon-inducible protein 16 (IFI16) and immunoglobulin mu heavy chain (IgM), alongside proteins involved in oxidative stress and signal transduction. Clinically, elevated circulating IFI16 correlates with RF/anti-CCP seropositivity and lung involvement in RA (Alunno et al. 2016). Similarly, IgM remains a diagnostic cornerstone as part of classical rheumatoid factor (IgM-RF) (van Boekel et al. 2002).

Immunogenicity screening further identified nine proteins harboring experimentally validated epitopes (Fig. S1c,d), including myosin-9 (MYH9), glutathione S-transferase P (GSTP1), and hemoglobin subunit gamma-1/2 (HBG1/2). Among them, MYH9 has previously been associated with RA disease activity and synovial aggressiveness (Lee et al. 2023), whereas HBG1/2 may represent novel candidate autoantigens in RA. In addition, 119 proteins showed predicted immunogenicity, with IFI16 and Apolipoprotein A-IV (APOA4) emerging as notable candidates (Fig. S1e). IFI16 and its isoform showed strong RA-specific upregulation in serum, recurrent elevation across multiple ADs in SPAID cross-disease analysis, and prior reports of anti-IFI16 antibodies in RA. Among them, the autoantigenic potential of IFI16 and its isoforms is well-established. Their prominent upregulation in RA serum is consistently replicated across multiple ADs in the SPAID cross-disease analysis, aligning with previous literature reports of anti-IFI16 antibodies in RA clinical cases (Alunno et al. 2016). In addition, APOA4 represents a completely novel candidate. While currently unlinked to rheumatoid arthritis in existing literature, APOA4 displayed highly specific enrichment in our RA cohorts alongside strong predicted immunogenicity, highlighting its unique value as a newly discovered immune target.

Overall, this case study demonstrates that SPAID successfully identifies known clinical biomarkers while helping discover novel candidates in ADs.

## Discussion

In this study, SPAID establishes a comprehensive resource to map the landscape of candidate autoantigens across various ADs. By organizing evidence within a structured two-level framework, SPAID significantly expands the known autoantigen landscape to encompass both canonical and non-canonical proteins. Compared with existing resources, the platform offers extensive functional annotations, ranging from macromolecular 3D structures to predicted PTM sites (Table 2). These rich architectural features facilitate a deeper mechanistic interpretation of antigen processing and epitope exposure (Curran et al. 2023). Furthermore, the practical utility of this framework is underscored by our validation in an independent RA cohort, which successfully recaptured established biomarkers and uncovered novel candidate autoantigens.

**Table 2:** Comparison of features among SPAID, Autoimmune Disease Database, AAgAtlas and PGG.MHC

Features	SPAID	Autoimmune Disease Database	AAgAtlas	Autoimmune Disease Explorer	PGG.MHC
Total AD-associated proteins	33,873	> 5,000	1,126	-	-
Genes	11,368	> 5,000	1,126	-	2,181 HLA alleles
Total proteomics samples for analysis	675	-	-	-	-
Validated epitopes (validated level)	21,565	-	-	-	-
MS-identified proteins (proteomics-based level)	26,085	-	-	-	-
Epitope-associated proteins	21,349	-	-	-	-
Protein annotation	√	√	√	×	×
Candidate autoantigen annotation	√	×	√	×	×
Gene annotation	√	√	√	×	×
Non-canonical Proteins included	√	×	×	×	×
Disease annotation	√	√	√	√	×

Although SPAID provides a comprehensive platform, several limitations should be noted. The current version restricts its immunogenicity predictions to HLA class I molecules and lacks HLA class II data, potentially missing key CD4 + T-cell-related autoantigens (Wucherpfennig et al. 2009). These predictions do not perfectly mirror in vivo antigen processing or immune recognition and require experimental verification. Additionally, the integrated proteomic datasets are largely derived from accessible peripheral tissues such as blood and skin, leaving tissue-specific autoantigens from less accessible organs underrepresented. Although MS effectively confirms the detection of a protein, it remains insufficient for characterizing its cellular localization, quantitative abundance, or biological relevance. Even with stringent false discovery rate control, mass spectrometry data alone cannot exclude false positives or non-functional translation products (Nesvizhskii 2014). Since the physiological presentation of non-canonical epitopes remains uncertain, these non-canonical proteins warrant cautious interpretation. Because these factors can present barriers to clinical translation, candidate autoantigens will require further functional and regulatory validation. Accordingly, SPAID serves as a candidate discovery resource rather than an absolute catalog of validated antigens.

While fully overcoming these systemic limitations remains an ongoing challenge, we aim to progressively address these limitations in future updates. Beyond expanding data modalities to include HLA class II profiles, future versions will integrate broader tissue proteomics. Our long-term commitment includes continuous infrastructure scaling to optimize the storage and interactive visualization of newly generated data, ensuring that SPAID remains a sustained, high-utility resource for the global research community.

## Supplementary Information

Supplementary Material 1. Figure S1: Application of SPAID leads to the discovery of biomarkers.

Supplementary Material 2. Table S1: Proteomic datasets included in SPAID.

Supplementary Material 3. Table S2: Predicted immunogenic MS peptides validated by MHC ligands.

Supplementary Material 4. Table S3: Differentially expressed proteins in Rheumatoid Arthritis.

## Data Availability

All processed data can be freely retrieved from the SPAID database at https://spaid.renlab.cn. The mass spectrometry proteomics data have been deposited to the ProteomeXchange Consortium via the PRIDE partner repository with the dataset identifier PXD078759.
