# Supplementary material for: SPAID: a comprehensive database for disease-specific autoantigens in autoimmune disorders
Source: Adv Biotechnol (Singap). 2026 Jun 8;4(2):23. doi: 10.1007/s44307-026-00117-8 (PMC13246995; doi:10.1007/s44307-026-00117-8)
Supplement: Supplementary file 1 — Supplementary Material 1. Figure S1: Application of SPAID leads to the discovery of biomarkers. [file 44307_2026_117_MOESM1_ESM.docx]

**
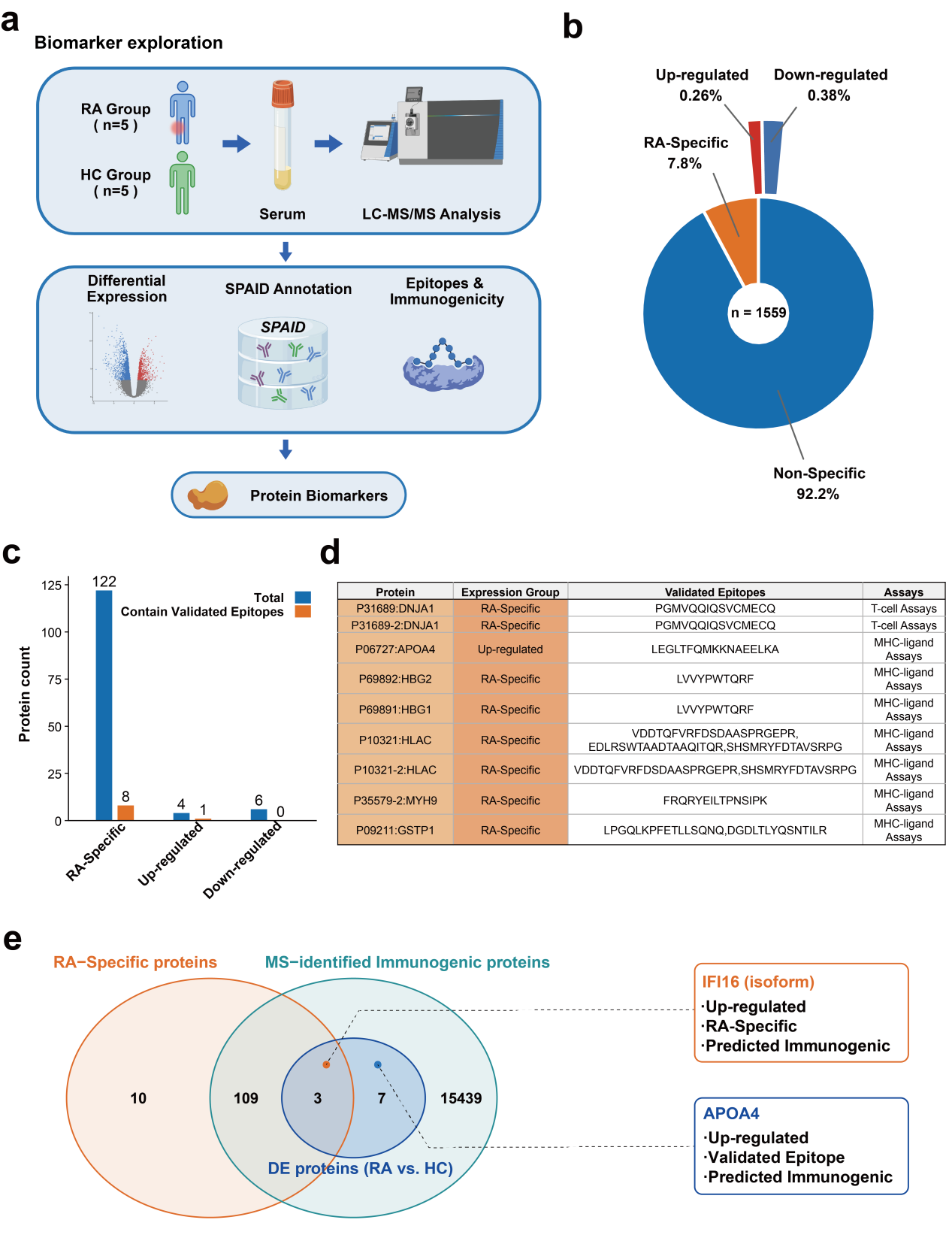
**

**Fig. S1. Application of SPAID leads to the discovery of biomarkers.** a: Workflow for biomarker exploration in RA through SPAID-based annotation. b: Distribution of protein expression patterns in RA cohort (RA vs HC). Red indicates up-regulated proteins, blue indicates down-regulated proteins, orange indicates RA-specific proteins, and light blue indicates non-specific proteins. c: The number of RA-specific and differentially expressed proteins, together with the number of proteins containing experimentally validated epitopes. Light blue bars indicate the total proteins in each category, whereas orange bars represent proteins containing validated epitopes. d: Nine proteins with at least one experimentally validated epitope and their corresponding epitope information, comprising one up-regulated protein and eight RA-specific proteins. e: Venn diagram illustrating the overlap among RA-specific proteins, MS-identified immunogenic proteins, and differentially expressed proteins, highlighting key candidate biomarkers such as IFI16 and APOA4.
